# The relationship between health-related quality of life and melancholic depressive symptoms is modified by brain insulin receptor gene network

**DOI:** 10.1038/s41598-021-00631-w

**Published:** 2021-11-03

**Authors:** Jannica S. Selenius, Patricia P. Silveira, Minna Salonen, Hannu Kautiainen, Mikaela von Bonsdorff, Eero Kajantie, Jari Lahti, Johan G. Eriksson, Niko S. Wasenius

**Affiliations:** 1 Folkhälsan Research Center, Finbyntie 136 Karjaa, 10300 Helsinki, Finland; 2 Department of General Practice and Primary Health Care, University of Helsinki and Helsinki University Hospital, Helsinki, Finland; 3 Department of Psychiatry, Faculty of Medicine, McGill University, 6875 Boulevard LaSalle, Verdun, QC H4H1R3 Canada; 4 Ludmer Centre for Neuroinformatic and Mental Health, Douglas Mental Health University Institute, McGill University, 6875 Boulevard LaSalle, Verdun, QC H4H1R3 Canada; 5 Public Health Promotion Unit, The National Institute for Health and Welfare, Helsinki, Finland; 6 Gerontology Research Center and Faculty of Sport and Health Sciences, University of Jyväskylä, Jyväskylä, Finland; 7 National Institute for Health and Welfare, Public Health Promotion Unit, Helsinki, Oulu, Finland; 8 PEDEGO Research Unit, MRC Oulu, Oulu University Hospital and University of Oulu, Oulu, Finland; 9 Children’s Hospital, Helsinki University Hospital and University of Helsinki, Helsinki, Finland; 10 Department of Clinical and Molecular Medicine, Norwegian University of Science and Technology, Trondheim, Norway; 11 Department of Psychology and Logopedics, University of Helsinki, P.O. Box 63, 00014 Helsinki, Finland; 12 Turku Institute for Advanced Studies, University of Turku, 20014 Turku, Finland; 13 Department of Obstetrics and Gynecology and Human Potential Translational Research Program, Yong Loo Lin School of Medicine, National University of Singapore, Singapore, Singapore; 14 Singapore Institute for Clinical Sciences (SICS), Agency for Science, Technology and Research (A*STAR), Singapore, Singapore

**Keywords:** Depression, Quality of life, Diabetes

## Abstract

To investigate whether expression-based polygenic risk scores for the insulin receptor gene network (ePRS-IRs) modifiy the association between type of depressive symptoms and health-related quality of life (HRQoL). This cross-sectional study includes 1558 individuals from the Helsinki Birth Cohort Study. Between 2001 and 2004, the Short Form-36 questionnaire was employed to assess mental and physical components of HRQoL and Beck Depression Inventory (BDI) to assess depressive symptoms. Depressive symptoms were categorized into minimal (BDI < 10), non-melancholic and melancholic types of depression. The ePRS-IRs were calculated for the hippocampal (hePRS-IR) and the mesocorticolimbic (mePRS-IR) regions of the brain. General linear regression models adjusted for age, sex, population stratification, lifestyle factors and body mass index were applied to analyze the data. Both types of depressive symptoms were associated with lower HRQoL (p < 0.0001). HePRS-IR modified the association between the types of depression and mental HRQoL (p for interaction = 0.005). Melancholic type of depressive symptoms was associated with higher mental HRQoL compared to the non-melancholic symptoms among individuals with low hePRS-IR (adjusted mean 4.1, 95% CI 0.7–7.4, p = 0.018). However, no such difference was evident in moderate or high hePRS-IR groups as higher hePRS-IR was associated with lower mental HRQoL (B = − 3.4, 95% CI − 5.6 to − 1.2) in individuals with melancholic type of depressive symptoms. No direct associations were detected between the ePRS-IRs and type of depressive symptoms or HRQoL. Variations in the glucose-insulin metabolism can lower HRQoL in individuals with melancholic depressive symptoms.

## Introduction

The demographic pyramid is changing and as healthcare and technology advances, humans live longer. However, the prevalence of chronic illnesses originating from unhealthy lifestyles are rapidly growing and as people’s life expectancy increases, the ageing population are faced with inevitable physical as well as mental and social challenges, ultimately affecting their health-related quality of life (HRQoL). For example, studies have reported that poorer HRQoL is commonly seen in individuals with diabetes^1–3^ and depression^4^. In depressed individuals, greatest reduction in HRQoL is found in those suffering from non-melancholic type of depressive symptoms^5^.

Depression is often long-lasting and lowers social functioning, working ability and overall HRQoL. Depression has a high comorbidity and is strongly associated with a range of conditions including insulin resistance and metabolic syndrome^6,7^. Previous studies have shown the importance of distinguishing between types of depression. Non-melancholic depression, characterized by mood reactivity, hypersomnia and weight gain, is associated with metabolic and inflammatory diseases^8–10^ more than melancholic type of depression. Melancholic depression, characterized by anhedonia, insomnia and weight loss, seems to mainly affect the central nervous system and does not associate in the same way with systemic processes^10,11^.

Depression has been linked to changes in the brain^12^. Central changes include volumetric reduction of the hippocampus, amygdala, insula, and medial prefrontal cortex^13,14^, areas which are classically associated with memory, emotion regulation and higher cognitive function^15^. Functionally, aside from altering the levels of numerous neurotransmitters, depression has also been linked to disturbances in glucose metabolism^16^. While previously it was thought that the brain was insensitive to insulin, we now know that insulin receptors (IR) play a part in numerous processes in the brain, such as learning, memory and depression^17–19^. IRs are more expressed in neurons than in glia cells, and are present in several locations in the brain including the hypothalamus, hippocampus and cerebral cortex^20^.

The risk of developing depression is influenced by lifestyle choices, environment, and genetics^21^. One approach to evaluate the genetic risk, is to convert genetic data from genome wide association studies (GWASs) into polygenic risk scores (PRSs)^22^. PRSs are traditionally calculated as a weighted sum of discovery GWAS based effect sizes of significant single nucleotide polymorphisms (SNPs)^23^. The traditional PRSs overlook the fact that genes operate in networks and have tissue specific biological functions. To tackle this shortcoming, a recent study developed novel biologically informed genetic scores for mesocorticolimbic and hippocampal insulin receptor-related gene networks. The expression based, brain region specific PRS for the insulin receptor gene network (ePRS-IR) was more strongly associated with Alzheimer’s disease, addiction and childhood impulsivity than the traditional PRSs^24^.

Thus far studies have focused on the well-established bidirectional relationship between insulin resistance and depression^25^. Less is known about the function of the insulin receptor and its effect on depression and HRQoL. The aim of this study is to investigate whether individual differences in the expression of the insulin receptor gene network can modify the relationship between type of depressive symptoms and HRQoL by employing ePRS-IRs.

## Research design and methods

### Participants

The Helsinki Birth Cohort Study (HBCS) consists of 13,345 individuals born between 1934 and 1944 at the Helsinki University Central Hospital or the Helsinki City Maternity Hospital^26^. As children, they attended welfare clinics and by1971, they received a unique personal identification number, as did all individuals of the Finnish population^27^. A baseline clinical examination, involving 2003 randomly selected cohort members, was conducted between 2001 and 2004. After excluding individuals with missing information, 1939 had sufficient information on depressive symptoms and 1930 on HRQoL After deleting duplicates, multiallelic SNPs and ambiguous SNPs, a biologically informed ePRS for the insulin receptor was computed for 1558 of those individuals. The study follows the guidelines of the Declaration of Helsinki and was approved by the Ethics Committee of Epidemiology and Public Health of the Hospital District of Helsinki and Uusimaa and that of the National Public Health Institute, Helsinki. All participants gave a written, informed consent.

### Health-related quality of life (HRQoL)

HRQoL was assessed using the validated 36-Item Short Form Health Survey (SF 36) version 1.0 questionnaire^28^. The SF 36 consists of the following eight domains: physical functioning (10 items), role limitations caused by physical health problems (4 items), role limitations caused by emotional health problems (3 items), bodily pain (2 items), general health (5 items), vitality (4 items), mental health (5 items) and social functioning (2 items). Scores for each item ranged from 0 (lowest perceived functioning) to 100 (highest perceived functioning). Based on these scores, physical and mental health component summary (PCS and MCS) scores were calculated using the US reference population (1990) to standardize the eight domains and for factor score coefficients. The summary scores were standardized using a mean of 50 and a standard deviation of 10. The psychometric properties of the Finnish SF-36 have been well validated^29^.The average age at the time of completing the questionnaire was 61.5 years (SD 2.9).

### Depression

Depression symptoms were screened for using the validated 21-item Beck Depression Inventory questionnaire (BDI). Each item was rated by the subjects from 0 to 3 according to how they felt at the moment. Items were summarized as a total BDI score, which can vary between 0 and 63. Scores 10 or higher were considered to indicate depression, as previously suggested^30^. The subjects with increased depressive symptoms were divided into melancholic and non-melancholic types of depression by comparing the means of summary scores of melancholic and non-melancholic items in BDI. Melancholic items were based on the Diagnostic and Statistical Manual for Mental Disorders (DSM-IV)-defined criteria^31^. Melancholic symptoms in the BDI included change of sleeping and appetite, feelings of guilt, irritability, loss of interest, loss of pleasure, past failure, punishment feelings and sadness^9,32–34^. Other items in BDI questionnaire were considered the non-melancholic. Subjects were classified to have melancholic type of depression if the mean of the melancholic summary scores were higher than that of the non-melancholic and vice versa. In the case of equal mean of summary scores, subjects were categorized into melancholic subgroups. The Finnish version of the BDI has been reported as reliable and well validated^35,36^.

### Covariates

At the baseline clinical examination, subjects answered questions about their current health situation and lifestyle characteristics. Anthropometrics including weight and height were measured with stadiometers (KaWe) and medical scales (SECA alpha 770), respectively. BMI was calculated as weight in kilograms divided with height in meters squared. Systolic and diastolic blood pressure was measured by a mercury sphygmomanometer from the right arm while the subject was sitting and was recorded as the mean of two successive readings. Smoking was coded as never, former and current, and alcohol use was coded as never or having quit, less than once a week or weekly. Highest attained socioeconomic status, obtained from Statistics Finland, was coded as high official, low official, self-employed and manual workers based on the original classification system^37^. The cohort members’ past 12-month leisure-time physical activity was assessed using a validated leisure-time physical activity (LTPA) questionnaire; the Kuopio Ischemic Heart Disease Risk Factor Study (KIHD)^38^. Leisure-time physical activity was measured in metabolic equivalents of task^39^, which were multiplied with time (hours) and frequency to calculate MET-hours, as previously suggested^40^. Cohort members were also asked using validated questionnaires about chronic diseases and conditions, including diabetes, cardiovascular conditions (congestive heart failure, arrhythmias, claudication, angina pectoris, previous heart attack and stroke), lung diseases (asthma, emphysema and chronic bronchitis), musculoskeletal disorders (rheumatoid arthritis, osteoporosis) and presence of cancer. The presence of comorbidities was coded as none, one or two or more. Blood samples were obtained to measure levels of total cholesterol, high-density lipoprotein (HDL) cholesterol and triglycerides. A standardized 2-h 75 g oral glucose tolerance test (OGTT) was performed and the World Health Organization (WHO) 2006 criteria^41^, self-reported, register linkage or usage of medication for diabetes were applied for diagnosing diabetes. Individuals who met the WHO 2006 criteria for impaired glucose tolerance or impaired fasting glucose were grouped together and called prediabetes. Glucose and insulin levels were measured at fasting as well as at 30 min and 2 h. Insulin resistance was determined by the homeostasis model assessment (HOMA), calculated by the formula: (fasting glucose x fasting insulin)/22.5^42^.

### Polygenic risk score (PRS)

Genotyping and ePRS-IR calculation was performed as previously described^24^. DNA was extracted from blood samples and genotyping was performed with the modified Illumina 610 k chip by the Wellcome Trust Sanger Institute, Cambridge, UK, according to standard protocols. Genomic coverage was extended by imputation using the 1000 Genomes Phase I integrated variant set (v3/April 2012; NCBI build 37/hg19) as the reference sample and IMPUTE2 software. Before imputing, quality control filters were applied. Specifically, SNP clustering probability for each genotype was set at > 95%, call rate at > 95% for individuals and markers (99% for markers with minor allele frequency (MAF) < 5%), MAF was set at > 1%, and the p value for the Hardy–Weinberg Equilibrium exact test p > 1 × 10^–6^. Moreover, heterozygosity, gender check and relatedness checks were performed and any discrepancies removed. The total number of SNPs in the imputed data was 39282668.

For the ePRS calculation, lists of genes co-expressed with the insulin receptor in the mesocorticolimbic system or hippocampus were created. In the original work^24^, a polygenic risk score for the insulin receptor (PRS-IR) was calculated using SNPs from these gene networks and the association betas from a discovery GWAS (ADHD in the mesocorticolimbic score^43^ and Alzheimer in the hippocampal score^44^). In the current study on HBCS, instead of weighing the SNPs by the association with ADHD or Alzheimer, these linkage disequilibria clumped SNPs were weighted with the betas from the Genotype-Tissue Expression (GTeX), a resource database and tissue bank for examining the relationship between genetic variation and gene expression in human tissues. The final lists of SNPs included 16,556 independent functional SNPs for mesocorticolimbic ePRS (from 263 genes) and 30,652 SNPs (from 498 genes) for the hippocampal ePRS. Final ePRSs were obtained by summation over all SNPs accounting for the sign of correlation coefficient between the genes and insulin receptor gene expression in the different regions. Thus, variations in the score used in the current study represent variations in the expression of the insulin receptor gene network in the specific brain region. In other words, a higher score indicates a higher expression of the insulin receptor gene network. The selection of the SNPs within a given clumping window was based on the lowest p-value. As a result, biologically informed mesocorticolimbic (mePRS-IR) and hippocampal (hePRS-IR) specific insulin receptor polygenic risk scores were calculated. For the analyses the PRS-IRs were standardized and reported both as a continuous and a categorical variable (0 = low =  < − 0.5 SD, 1 = moderate = − 0.5 to 0.5 SD, and 2 = high =  > 0.5 SD).

### Statistical analysis

The data are reported in means (standard deviation or 95% confidence intervals), medians (interquartile range) or in counts (percentage). The baseline characteristics were analysed with analysis of variance for continuous variables and chi-square test for categorical variables. General linear models (GLM) allowed us to assess the association between components of HRQoL, types of depression, mePRS-IR, hePRS-IR. GLM were also applied to investigate the depression type by PRS-IRs interaction effect on HRQoL. The regression models were adjusted for age, sex, smoking, alcohol usage, socioeconomic status (SES), presence of chronic diseases, BMI, LTPA and population stratification^45,46^, as well as for significant interactions between covariates and depression types on HRQoL when applicable. In the case of not meeting the assumptions (e.g. non-normal distribution) bootstrap style analyses (10,000 replications) were applied when analysing the baseline characteristics as well as when applying general linear models. Multinomial logistic regression with bootstrap style analyses (1500 repetitions) was applied to investigate the association between the depression subgroups. Significance level of 0.005 was applied when testing associations between the hePRS-IRs and subgroups of HRQoL. In all other analyses p value of 0.05 was used for statistical significance. Statistical analyses were carried out using Stata/MP version 16.1 (Stata Corporation, College Station, TX, USA).

## Results

Our study comprised 1558 subjects, of which 876 (56.2%) were women. The mean age of the participants at the time of the examination was 61.5 years (SD 2.9 for all). Table 1 shows the characteristics of the cohort members according to type of depressive symptoms. Individuals with symptoms of non-melancholic depression suffered more frequently from multiple chronic disease and had higher BMI and blood pressure than individuals with normal BDI score or melancholic type of depressive symptoms.

**Table 1:** Baseline characteristics according to depressive type.

Characteristics	Depressive type			P for between group comparison
	BDI < 10 (n = 1262)	Non-melancholic (n = 199)	Melancholic (n = 97)	
Female, n (%)	674 (53)	151 (76)	51 (53)	< 0.001
Age (years), mean (sd)	61.4 (2.8)	62.2 (3.3)	61.6 (3.0)	0.007
**Alcohol use**				0.001
Never or quit earlier	75 (6)	23 (12)	15 (15)	
Less than once a week	537 (43)	92 (46)	38 (39)	
Weekly	650 (51)	84 (42)	44 (45)	
Smoking				0.10
Never	549 (44)	81 (41)	35 (36)	
Former	425 (34)	57 (29)	36 (37)	
Current	288 (23)	61 (31)	26 (27)	
**Chronic diseases**				< 0.001
None	423 (34)	39 (20)	21 (22)	
1 disease	418 (33)	57 (29)	24 (25)	
≥ 2 diseases	421 (33)	103 (52)	52 (54)	
**Socioeconomic status**				0.006
High official	203 (16)	15 (8)	8 (8)	
Low official	531 (42)	101 (51)	42 (43)	
Self-employed	119 (9)	22 (11)	8 (8)	
Manual worker	409 (32)	61 (31)	39 (40)	
Body mass index (kg/m2)	27.4 (4.4)	29.2 (5.6)	27.5 (4.8)	< 0.001
Systolic BP (mmHg )	145.9 (20.5)	148.3 (19.7)	138.9 (18.6)	< 0.001
Diastolic BP (mmHg )	89.3 (10.5)	89.4 (10.5)	85.4 (8.7)	< 0.001
Triglycerides (mmol/l ), median (iqr)	1.3 (1, 1.8)	1.3 (1, 2)	1.3 (1, 1.7)	0.08
Total cholesterol (mmol/l )	5.9 (1.0)	6.0 (1.0)	5.8 (1.3)	0.40
HDL-cholesterol (mmol/l )	1.6 (0.4)	1.6 (0.4)	1.6 (0.5)	0.96
Fasting glucose (mmol/l )	5.8 (1.2)	6.0 (1.7)	6.0 (1.9)	0.21
30 min glucose (mmol/l )	9.4 (2.2)	9.6 (2.3)	9.6 (2.3)	0.55
2 h glucose (mmol/l )	7.8 (3.3)	8.4 (4.0)	8.1 (3.8)	0.09
Fasting insulin (mU/l )	8.0 (5.5, 12.4)	9.2 (5.7, 14.7)	8.1 (5.1, 11.2)	0.12
30 min insulin (mU/l )	58 (38.2, 87.3)	63.4 (44, 90.1)	54.9 (33.2, 88.3)	0.74
2 h insulin (mU/l )	56.8 (36.6, 95.1)	69.5 (42.6, 105.2)	50.5 (32.2, 70.1)	0.06
HOMA-IR ()	2.0 (1.3, 3.3)	2.3 (1.4, 3.9)	2.0 (1.2, 3.4)	0.17
**Diabetes status**				0.15
Normoglyceamia	674 (53.4)	93 (46.7)	47 (48.5)	
Prediabetes	390 (30.9)	62 (31.2)	35 (36.1)	
Diabetes	198 (15.7)	44 (22.1)	15 (15.5)	
LTPA (METh/wk)	35.7 (19.7, 59.1)	33.6 (17.2, 61.1)	35.5 (22.6, 67)	0.32

*BDI* Beck Depression Inventory, *BP* blood pressure, *HDL* high-density lipoprotein, *HOMA* homeostasis model assessment for insulin resistance, *LPTA* leisure time physical activity, *MET* metabolic equivalent of task (1 MET = 3.5 ml of O_2_/kg/min). P values of the continuous variables were based on the bootstrap style method of 10,000 repetitions in all variables except for systolic BP, diastolic BP, and HDL-cholesterol.

Both non-melancholic and melancholic depression were associated with lower HRQoL (p < 0.001) (Table 2). A nominally significant association was found between the general health and hePRS-IR (p = 0.04). However, after correcting for multiple testing this association was no longer (Bonferroni corrected p value = 0.40) and no association was found between the hePRS-IR or mePRS-IR and the eight items of HRQoL, MCS, and PCS (Table 3) or type of depression (Table 4, 5). The hePRS-IR was associated with 2 h glucose and insulin concentrations in the OGTT (p values = 0.02 and 0.002, respectively; Table 6), but not with HOMA.

**Table 2:** Association between the physical and mental components of health-related quality of life (HRQoL) and type of depressive symptoms.

		BDI < 10	Non-melancholic	Melancholic	p, adjusted
PCS, mean (SD)		49.2 (7.9)	**42.1 (10.1)**	45.8 (9.7)	< 0.001
MCS, mean (SD)		56.4 (6.1)	**43.3 (11.6)**	**44.6 (11.7)**	< 0.001

Model adjusted for age, sex and population stratification, smoking, alcohol usage, socioeconomic class, body mass index, leisure-time physical activity and comorbidities.

*BDI* Beck Depression Inventory, *PCS* physical component score, *MCS* mental component score, *SD* standard deviation. P values were based on the bootstrap style method of 10,000 repetitions.

**Table 3:** The association between the biologically informed polygenic risk score for insulin receptor (ePRS-IR) and the physical component (PCS) and the mental component (MCS) of health-related quality of life (HRQoL) as well as the domains of the SF-36.

	Crude				Fully adjusted			
	B	95% CI		p	B	95% CI		p
**hePRS-IR**								
Physical functioning	− 0.61	− 1.46	0.25	0.16	0.42	− 0.43	1.26	0.34
General health	− 0.82	− 1.72	0.08	0.08	0.90	0.03	1.78	0.04
Vitality	− 0.68	− 1.61	0.25	0.15	0.47	− 0.50	1.43	0.34
Mental health	− 0.25	− 0.98	0.47	0.50	0.43	− 0.34	1.21	0.28
Physical role functioning	0.35	− 1.25	1.95	0.67	0.43	− 1.10	1.97	0.58
Emotional role functioning	− 0.32	− 1.81	1.17	0.67	0.77	− 0.71	2.25	0.31
Social role functioning	− 0.07	− 0.97	0.83	0.88	0.46	− 0.44	1.35	0.32
Bodily pain	− 0.49	− 1.59	0.60	0.38	0.78	− 0.27	1.83	0.15
MCS	− 0.12	− 0.56	0.31	0.59	− 0.12	− 0.57	0.30	0.59
PCS	− 0.20	− 0.63	0.21	0.35	− 0.08	− 0.46	0.32	0.71
**mePRS-IR**								
Physical functioning	− 0.24	− 1.00	0.51	0.53	0.12	− 0.64	0.87	0.76
General health	− 0.58	− 1.39	0.23	0.16	0.66	− 0.13	1.46	0.10
Vitality	− 0.55	− 1.45	0.35	0.23	0.33	− 0.60	1.27	0.49
Mental health	− 0.22	− 0.94	0.49	0.54	0.41	− 0.36	1.17	0.30
Physical role functioning	0.61	− 0.95	2.17	0.44	0.24	− 1.24	1.71	0.75
Emotional role functioning	− 0.19	− 1.66	1.28	0.80	0.66	− 0.80	2.12	0.38
Social role functioning	0.03	− 0.84	0.91	0.94	0.36	− 0.51	1.23	0.42
Bodily pain	− 0.39	− 1.45	0.67	0.47	0.67	− 0.35	1.69	0.20
MCS	0.23	− 0.22	0.69	0.33	0.22	− 0.23	0.69	0.34
PCS	0.25	− 0.16	0.66	0.24	0.13	− 0.23	0.53	0.49

Model 1 adjusted for age, sex and population stratification. Model 2 adjusted for Model 1 + smoking, alcohol usage, socioeconomic status, comorbidities, body mass index, leisure-time physical activity and comorbidities.

*PF* physical functioning, *GH* general health, *VT* vitality, *MH* mental health, *RP* physical role functioning, *RE* emotional role functioning, *SF* social role functioning, *BP* bodily pain. *hePRS-IR* hippocampal biologically informed polygenic risk score for insulin receptor, *mePRS-IR* mesocorticolimbic biologically informed polygenic risk score for insulin receptor, *B* regression coefficient. P values were based on the bootstrap style method of 10,000 repetitions.

**Table 4:** The biologically informed hippocampal (hePRS-IR) and mesocorticolimbic (mePRS-IR) polygenic risk score for insulin receptor according to depressive type.

Variable	PRS-IR groups			p-value		
	< −0.5 SD	− 0.5 to 0.5 SD	> 0.5 SD	− 0.5 to 0.5 SD vs < − 0.5 SD	> 0.5 SD vs < − 0.5 SD	> 0.5 SD vs − 0.5 to 0.5 SD
**hePRS-IR**						
BDI < 10, %	81.3 (1.9)	79.9 (1.7)	82.1 (1.7)	0.57	0.75	0.35
Non-melancholic, %	12.1 (1.6)	13.2 (1.4)	12.8 (1.5)	0.62	0.75	0.86
Melancholic, %	6.5 (1.3)	6.9 (1.1)	5 (1.1)	0.82	0.35	0.2
**mePRS-IR**						
BDI < 10, %	81.8 (2.1)	80.6 (1.8)	80.5 (2.3)	0.65	0.67	0.98
Non-melancholic, %	12.1 (1.5)	11.7 (1.3)	14.9 (1.7)	0.87	0.2	0.15
Melancholic, %	6.1 (1.6)	7.6 (1.3)	4.6 (1.9)	0.43	0.54	0.17

Data are shown as predicted mean probability (standard error)**.** Model adjusted for age, sex and population stratification, smoking, alcohol usage, socioeconomic class, body mass index, leisure-time physical activity and comorbidities.

*BDI* Beck Depression Inventory, *hePRS-IR* hippocampal biologically informed polygenic risk score for insulin resistance, *mePRS-IR* mesocorticolimbic biologically informed polygenic risk score for insulin resistance, *SD* standard deviation. P values were based on the bootstrap style method of 1500 repetitions.

**Table 5:** The association between the biologically informed polygenic risk score for insulin receptor (ePRS-IR) and non-melancholic and melancholic depression with ePRS-IR as continuous variable.

	B	95% CI		p
**Non-melancholic depression**				
hePRS-IR				
Crude	0.06	− 0.10	0.21	0.48
Adjusted	0.03	− 0.12	0.19	0.67
mePRS-IR				
Crude	− 0.02	− 0.18	0.15	0.85
Adjusted	0.01	− 0.16	0.17	0.95
**Melancholic depression**				
hePRS-IR				
Crude	− 0.13	− 0.35	0.07	0.22
Adjusted	− 0.13	− 0.35	0.08	0.25
mePRS-IR				
Crude	− 0.08	− 0.28	0.12	0.40
Adjusted	− 0.09	− 0.29	0.12	0.40

Model 1 = adjusted for age, sex and population stratification. Model 2 = adjusted for Model 1 + smoking, alcohol usage, socioeconomic class, body mass index, leisure-time physical activity and comorbidities. *hePRS-IR* hippocampal biologically informed polygenic risk score for insulin receptor, *mePRS-IR* mesocorticolimbic biologically informed polygenic risk score for insulin receptor, *B* regression coefficient. P values were based on the bootstrap style method of 1500 repetitions.

**Table 6:** The association between the hePRS-IR and mePRS-IR on markers of glucose and insulin metabolism and insulin resistance.

Variable	Crude			Adjusted		
	B	95% CI	P	B	95% CI	p
**Fasting glucose (mmol/l)**						
hePRS-IR	0.04	(− 0.02 to 0.11)	0.211	0.03	(− 0.04 to 0.09)	0.407
mePRS-IR	− 0.02	(− 0.09 to 0.05)	0.587	0.01	(− 0.05 to 0.07)	0.689
**30 min glucose (mmol/l)**						
hePRS-IR	0.13	(0.02 to 0.24)	0.023	0.1	(− 0.01 to 0.2)	0.067
mePRS-IR	− 0.07	(− 0.19 to 0.04)	0.216	− 0.03	(− 0.14 to 0.08)	0.574
**2 h glucose (mmol/l)**						
hePRS-IR	0.26	(0.09 to 0.43)	0.003	0.19	(0.04 to 0.35)	0.02
mePRS-IR	− 0.04	(− 0.22 to 0.15)	0.687	0.02	(− 0.15 to 0.2)	0.819
**Fasting insulin (mU/l)**						
hePRS-IR	0.53	(0.14 to 0.88)	0.005	0.31	(− 0.02 to 0.61)	0.056
mePRS-IR	− 0.15	(− 0.61 to 0.3)	0.526	0.05	(− 0.42 to 0.46)	0.809
**30 min insulin (mU/l)**						
hePRS-IR	2.45	(− 0.08 to 4.89)	0.054	1.44	(− 0.98 to 3.82)	0.243
mePRS-IR	− 0.79	(− 3.01 to 1.59)	0.504	− 0.18	(− 2.43 to 2.09)	0.875
**2 h insulin (mU/l)**						
hePRS-IR	6.31	(3.3 to 9.56)	< 0.001	4.64	(1.9 to 7.79)	0.002
mePRS-IR	− 1.72	(− 4.68 to 1.51)	0.277	− 0.73	(− 3.58 to 2.53)	0.633
**HOMA**						
hePRS-IR	0.14	(0 to 0.26)	0.042	0.06	(− 0.07 to 0.17)	0.274
mePRS-IR	− 0.02	(− 0.17 to 0.13)	0.759	0.05	(− 0.09 to 0.18)	0.502

Model 1 = adjusted for population stratification. Model 2 = adjusted for Model 1 + smoking, alcohol usage, socioeconomic status body mass index, leisure-time physical activity and comorbidities. *hePRS-IR* hippocampal biologically informed polygenic risk score for insulin receptor, *mePRS-IR* mesocorticolimbic biologically informed polygenic risk score for insulin receptor, *B* unstandardized regression coefficient. Confidence intervals and P values were based on the bootstrap style method of 10,000 repetitions.

**Figure 1:**
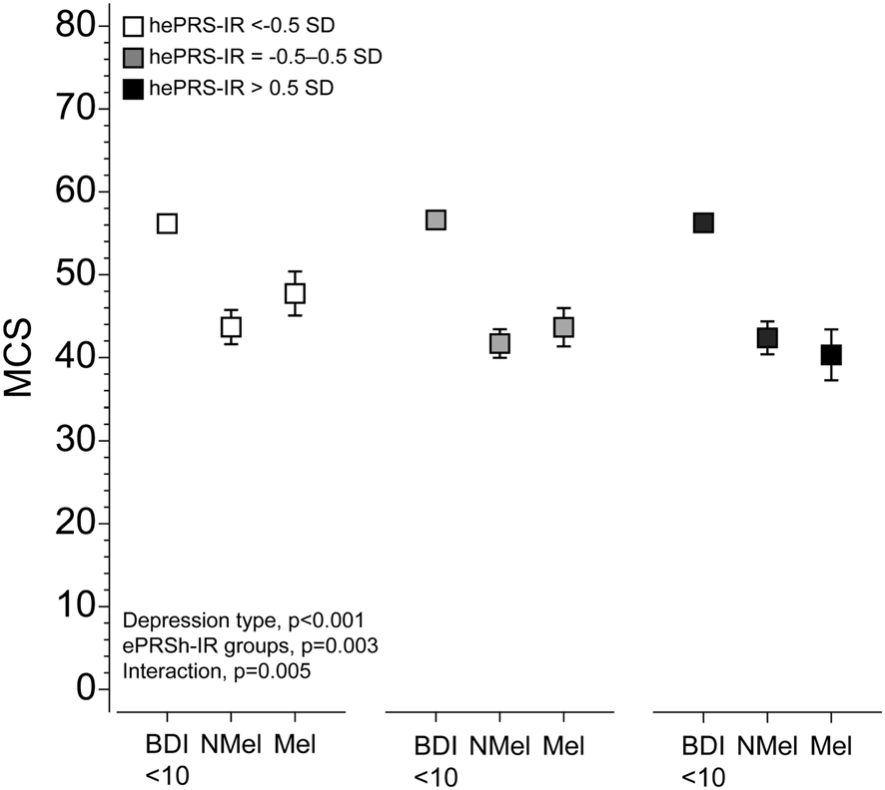
The association of the types of depression and mental component score of the SF-36 according to the high/medium/low scores of the biologically informed hippocampal polygenic risk score for the insulin receptor gene network (hePRS-IR). Model adjusted for age, sex and population stratification, smoking, alcohol usage, socioeconomic class, body mass index, leisure-time physical activity and comorbidities. *MCS* mental component score of the SF-36, *hePRS-IR* biologically informed hippocampal polygenic risk score for insulin receptor, *SD* standard deviation, *BDI* Beck Depression Inventory, *BDI < 10* no depression, *NMEL* non-melancholic depression, *Mel* Melancholic depression.

**Figure 2:**
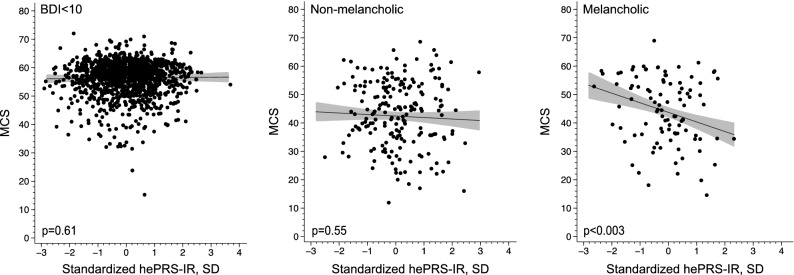
Association between the biologically informed hippocampal polygenic risk score for the insulin receptor gene network (hePRS-IR) and mental component score of the SF-36 (MCS) in individuals with no, non-melancholic or melancholic depressive symptoms. Confidence intervals and P-values were based on the bootstrap style method of 10,000 repetitions. (**a**) BDI < 10. (**b**) Non-melancholic depressive symptoms. (**c**) Melancholic depressive symptoms. Model adjusted for age, sex and population stratification, smoking, alcohol usage, socioeconomic class, body mass index, leisure-time physical activity and comorbidities. *MCS* mental component score of SF-36, *BDI* Beck Depression Inventory, *hePRS-IR* biologically informed hippocampal polygenic risk score for insulin receptor, *SD* standard deviation.

Figures 1 and 2 demonstrate the significant interaction between type of depressive symptoms and hePRS-IR on MCS when analysing hePRS-IR as categorical variable (p for interaction = 0.005), respectively as a continuous variable (p for interaction = 0.008). Regardless of the he-PRS-IR status, both non-melancholic and melancholic were associated with lower MCS compared to the BDI < 10 group (Fig. 1). However, in individuals with low hePRS-IR, melancholic depression was associated with higher MCS (4.1 95% CI 0.7–7.4, p = 0.018) compared to non-melancholic group. No such difference was detected among individuals with moderate or high hePRS-IR groups (p ≥ 0.18) (Fig. 1) as MCS decreased linearly in the melancholic group when applying either categorical (p < 0.001, Fig. 1) or continuous hePRS-IR (p = 0.003, Fig. 2).

Further adjustment for glucose regulation status did not substantially alter the found association or interactions (p for interaction = 0.009 for continuous hePRS-IR and p for interaction = 0.006 for categorical hePRS-IR).

Our analyses detected no significant association between type of depression by ePRS-IR interaction on PCS (p > 0.05) nor between type of depression and continuous or categorical mePRS-IR on MCS or PCS (p > 0.05).

## Discussion

Our findings indicate that higher hippocampal expression of the insulin receptor gene network is associated with lower mental HRQoL specifically in individuals with symptoms of melancholic depression, suggesting that individual differences in the function of the hippocampal insulin receptor gene network can possibly play a role in the relationship between type of depressive symptoms and HRQoL. No evidence of such modifying effect was found for the physical component of HRQoL nor by the mePRS-IR. This lowering effect decreased as the gene expression score decreased, and HRQoL did not distinguishably differ between melancholic and non-melancholic depressive type in individuals with low ePRS-IR. Lower ePRS-IR in itself showed no association with decreased HRQoL nor with any of the two types of depressive symptoms.

The hePRS-IR displayed a significant association with 2 h glucose and insulin concentrations in the OGTT. None of the ePRS-IRs were associated with HOMA. Thus, we showed that the ePRS-IRs measure what they were intended to when they were created, i.e. variations in the glucose insulin metabolism. As such, the application of the ePRSs allowed us to investigate the role of variations in insulin metabolism in specific tissues in individuals regardless of insulin resistance status.

Insulin is considered to be a cognitive modulator^47^ and disturbances in glucose and insulin metabolism are linked to several psychiatric conditions, including depression^16^. As established earlier, the ePRS-IRs measure variation in insulin glucose metabolism and not metabolic states such as insulin resistance. This could explain the lack of direct association between the ePRS-IRs and depressive symptoms. Both types of depression were associated with lowered HRQoL regardless of genetic markers for different expression of the insulin receptor gene network. However, individuals with melancholic, but not non-melancholic, depressive symptoms showed a clear descending trend in HRQoL with the hePRS-IR. Previous studies have shown an association between non-melancholic depression and peripheral insulin resistance and metabolic syndrome^10,11^, while brain insulin sensitivity has been shown to correspond with favourable body fat distribution and weight loss^48^. The lack of association between non-melancholic depressive symptoms and the ePRS-IRs is compliant with the ePRS-IRs not being associated with HOMA. Individuals with melancholic depression have been found to suffer less frequently from metabolic diseases associated with insulin resistance compared to non-melancholic depression^8^, which could explain the role of high expression of the insulin receptor gene network on HRQoL in our study.

HRQoL is lower among individuals with peripheral insulin resistance^49^. According to our results, HRQoL did not display direct association with either of the ePRS-IRs, which suggests that the insulin receptor gene network does not affect HRQoL the same way insulin resistance does. HRQoL has repeatedly been demonstrated to be lowered in depressed individuals^50^, as was the case in our study. Although the modification of the hePRS-IR was associated with further decreased HRQoL in the melancholic depressive subtype, both subtypes have significantly lower HRQoL compared to the individuals with BDI score < 10.

The modification by a high genetic risk score in depressed individuals was detected only by the hePRS-IR whereas the mePRS-IR did not affect HRQoL. Moreover, only the hePRS-IR was associated with worse glucose metabolism. Studies have suggested that changes in the hippocampal region of the brain play part in the pathophysiology of depression^13,14^. The mesocorticolimbic region of the brain has previously been considered to be associated primarily with other neuropsychiatric disorders such as ADHD and addiction^51^. In light of these studies, it can be argued that any modification by high expression of the insulin receptor gene network in depressed individuals would be observed in those with high hePRS-IR, as our results suggest.

The strengths of our study involve the use of the well-characterized HBCS and the use of both clinical and registered-based data in producing our results, as well as the validated SF-36 for assessing HRQoL and the standardized BDI questionnaire for assessing depression. Moreover, the biologically informed ePRS-IR employ whole gene networks instead of single genes, SNPs or discovery GWAS based PRSs to predict risk. However, our cohort is from a homogenous, restricted area in Finland and the findings from this study might therefore have to be cautiously implemented in other populations. Concerning the application of our findings, ePRSs are best utilized in combination with environmental and clinical risk factors when interpreting an individual’s lifetime risk of disease.

In conclusion, a high expression of the insulin receptor gene network is associated with lower mental HRQoL in individuals with symptoms of melancholic depression, suggesting that differences in the function of the insulin receptor share an association with the relationship between type of depressive symptoms and HRQoL.

## Data Availability

The data analyzed during the current study are available from the corresponding author on reasonable request.
